# Political Regime and Human Capital: A Cross-Country Analysis

**DOI:** 10.1007/s11205-011-9983-6

**Published:** 2012-03-20

**Authors:** Jeroen Klomp, Jakob de Haan

**Affiliations:** 1 Social Sciences Group, Wageningen University, PO Box 8130, 6700 EW Wageningen, The Netherlands; 2 Faculty of Economics and Business, University of Groningen, PO Box 800, 9700 AV Groningen, The Netherlands; 3 De Nederlandsche Bank, Amsterdam, The Netherlands; 4 CESifo, Munich, Germany

**Keywords:** Human capital, Political regime, Latent variable, Structural equation model

## Abstract

We examine the relationship between different dimensions of the political regime in place and human capital using a two-step structural equation model. In the first step, we employ factor analysis on 16 human capital indicators to construct two new human capital measures (basic and advanced human capital). In the second step, we estimate the impact of our political variables on human capital, using a cross-sectional structural model for some 100 countries. We conclude that democracy is positively related to basic human capital, while regime instability has a negative link with basic human capital. Governance has a positive relationship with advanced human capital, while government instability has a negative link with advanced human capital. Finally, we also find an indirect positive effect of governance and democracy on both types of human capital through their effect on income.

**Electronic supplementary material:**

The online version of this article (doi:10.1007/s11205-011-9983-6) contains supplementary material, which is available to authorized users.

Many studies have analyzed whether human capital is related to political institutions. There is substantive evidence supporting Lipset’s (1959) hypothesis that high educational standards are one of the basic conditions for sustaining democracy (see, for example, Castello-Climent 2008; Barro 1999; Acemoglu et al. 2005). However, the causal relationship may also run from political institutions to human capital (cf. Feng 2003; Ross 2006). The purpose of this paper is to examine whether political institutions affect the accumulation of human capital. This is an important issue, as several studies conclude that human capital is one of the main drivers of economic growth (see, for instance, Mankiw et al. 1992).^1^

Our paper is not the first to analyze this issue. Previous studies include Baum and Lake (2003), Lake and Baum (2001), Feng (2003) and Ross (2006) who all report a positive significant impact of some proxy for democracy on various human capital indicators. However, most studies analyzing the impact of political institutions on cross-country differences in human capital have various shortcomings. First, there is a measurement problem. Most studies employ school enrolment rates or average years of schooling as an indicator of human capital, thereby implicitly assuming that human capital is a one-dimensional concept.^2^ Similarly, studies employ political indicators often in a rather arbitrary way. Second, authors generally do not examine how sensitive their results are with respect to: (1) model specification, and (2) sample selection. This implies that a particular variable may be significant in one model specification, but can become insignificant in another model that may also be justified on theoretical grounds. Likewise, a variable may be significant in a model estimated for a particular group of countries but may become insignificant if a different sample is used.

To deal with these criticisms, we use a two-step structural equation model. In the first step, we apply factor analysis to 16 human capital indicators for 123 countries to examine whether human capital is multi-dimensional. It turns out that two factors capture most of the variance of these indicators. Using this result, we construct two measures of human capital (‘basic’ and ‘advanced’ human capital) to examine in the second step the impact of various dimensions of the political regime in place on human capital in a structural model with various economic and demographic control variables. We focus on different dimensions of the political regime in place: the type of regime (i.e., the level of democracy), the stability of the regime, and governance. We take a long list of potential control variables into account that have been suggested by previous studies. Using the general-to-specific approach (Campos et al. 2004), we decide on the specification of our structural model. After testing for the sensitivity of our results with respect to sample selection, we conclude that democracy is positively related to basic human capital, while regime instability has a negative link with basic human capital. Governance has a positive relationship with advanced human capital, while government instability has a negative link with advanced human capital. Finally, we also find an indirect positive link between governance and democracy and both types of human capital through the impact of democracy and governance on income.

The remainder of the paper is structured as follows. First, the literature on the relationship between human capital and political factors is reviewed. Second, factor analysis and our results for human capital and political factors are explained. Third, the structural equation model and our results are described. Fourth, the outcomes on the relationship between human capital and political factors are presented, followed by a sensitivity analysis. The last section offers our conclusions.

## Political Factors and Human Capital

On the basis of previous studies, we identify three dimensions of the political regime in place that may influence human capital: (1) the type of regime, (2) the stability of the regime, and (3) governance of the regime.

The first political factor we distinguish is the *type of regime* in place. There are several reasons why democratic societies may have better human capital then autocratic societies. First, democracies will spend more on education since autocracies generally rely on the rich, who care less about public spending on education than the poor or middle classes. Brown and Hunter (2000, 2004) find a significant relation between human capital formation measured by education spending and democracy in Latin America.^3^ Second, Feng (2003) argues that an educated population may threaten authoritarian regimes. For example, during the “cultural revolution” in China, schools were closed and graduates were sent to the countryside. Finally, greater government attention will be paid to education issues in democracies since failure to do so may result in politicians being removed from office (Ross 2006). Baum and Lake (2003), Lake and Baum (2001) and Feng (2003) report a positive and significant impact of democracy on various human capital indicators. However, Helliwell (1994) finds that the significant positive relationship between school enrolment and democracy becomes less strong and sometimes even insignificant when initial GDP is taken into account.

The second political factor that we distinguish is the *stability of the regime*. It is likely that individuals and governments are more willing to invest in human capital if the political environment is stable and therefore more certain. The demand for education and Research and Development (R&D) decreases in unstable periods as in such an environment it is often difficult to make rational calculations on the returns on investing in human capital. At the same time, political instability caused by, for example, riots, civil war or general strikes (in particular of education personnel) can disrupt the educational system in a country. Alternatively, individuals may prefer to concentrate their investments in human capital during more unstable political situations since human capital is a less specific asset and easier to transfer. However, most empirical evidence points in another direction. Katona (1980), McMahon (1987), and Francis (2007) report a significant negative relationship between human capital (proxied by enrollment rates) and internal conflict. Likewise, Maloney (2002) argues that the endemic political instability in Latin America may have been one of the major reasons why countries in the region have low levels of human capital. Fedderke and Klitgaard (1998) distinguish between “regime-threatening” and “non-regime threatening” political instability and find that only the former is negatively related to education levels.

The final political factor we distinguish is *governance*. The effectiveness of the government may play a role in explaining cross-country differences in human capital. For example, for R&D it is important to have a well functioning judicial system to secure patents. Likewise, endemic corruption and an inefficient bureaucracy may distort the allocation of the education budget. According to the theoretical model of Veira and Teixeira (2006), corruption causes lower levels of education due to a decrease in efficiency in a corrupted country. Bhattacharyya (2009) reports a positive relationship between the Rule of Law indicator of the International Country Risk Guide and the schooling data of Barro and Lee (2001).

## Factor Analysis: Method

As our aim is to estimate the impact of several political factors on human capital, we have to quantify (or measure) these political factors. One of the difficulties involved in incorporating political variables into an econometric analysis is how to measure various types of political events, systems, or concepts. While some political events are of a discrete nature (e.g., coups d’état) other concepts (e.g., democracy) are more difficult to quantify.

Studies that examine the effect of political factors, like democracy, usually choose their political indicators in a rather arbitrary way. According to Munck and Verkuilen (2002, 5–6), “with a few notable exceptions, quantitative researchers have paid sparse attention to the quality of the data on democracy that they analyze… To a large extent, problems of causal inference have overshadowed the equally important problems of conceptualization and measurement”. Treier and Jackman (2008, 202) argue that “Some researchers even operationalize democracy with a single indicator… However, the hope that a solitary indicator circumvents these measurement issues is illusory; indeed, most scholars agree that democracy is multifaceted, and hence not well characterized by a single indicator”. No doubt, the same applies to indicators of other dimensions of the political system. If authors employ more than one indicator, they generally do not examine whether the indicators used really capture the latent construct that they are supposed to represent. Furthermore, most indicators of political institutions contain measurement errors. In other words, there is an errors-in-variables problem, which causes biased estimation results (Treier and Jackman 2008; de Haan 2007; Dreher et al. 2007).

Likewise, studies that include human capital as a variable usually employ an arbitrarily chosen (one-dimensional) indicator, like initial years of schooling or school enrolment rates. However, it may be questioned whether these indicators represent all dimensions of human capital. Woodhall (1987, p. 219) defines human capital as “the process by which people—by means of education, training or other activities invest in themselves in hope of raising their future income”. This definition suggests that human capital is a multi-faceted concept. Furthermore, also most indicators of human capital contain some measurement error leading to a low quality data and biased estimation (Cohen and Soto 2007; Krueger and Lindahl 2001).

To come up with better measures that include more information and to determine whether human capital and political institutions have a multidimensional character, we employ a so-called explorative factor analysis (EFA). The objective of an EFA is to identify what different indicators of a latent variable (like human capital and political factors) have in common and to separate common factors from specific factors.^4^ Following Wansbeek and Meijer (2000) and Lattin et al. (2003), the EFA model can be written as:

1 $$ x_{i} = \Updelta \xi_{i} + \varepsilon_{i} $$

where *x*_*i*_ is a vector containing the *M* indicators for observation *i*, *i* = 1, …, *k* (in our case the indicators of human capital and the indicators of several dimensions of the political regime), ∆ is a vector of factor loadings of order *M* × *k*, and *ξ* is a vector of latent variables with mean zero and positive define covariance. The random error term *ε* is assumed to be uncorrelated with the latent variables.^5^ Under these assumptions, the covariance matrix of *x*_*i*_ is:

2 $$ \Upxi = \Updelta \Upphi \Updelta^{\prime } + \Upomega $$

where Ξ is the parameterized covariance matrix that can be decomposed in the covariance matrix of the factors Φ and the diagonal covariance matrix of error terms Ω. The model is estimated with the maximum likelihood (ML) method. The log-likelihood function can be written as:^6^

3 $$ \ln L = \ln \left| \Upxi \right| + tr[S\Upxi^{ - 1} ] $$

where *S* represents the sample covariance matrix. Minimizing this fit function means choosing the values for the unknown parameters that lead to the implied covariance matrix that is as close as possible to the sample covariance matrix.

The next step is to decide on the number of factors to represent human capital or political institutions on the basis of the scree plot, which plots the number of factors against the eigenvalues of the covariance matrix of the indicators. In general, there are two ways of interpreting the graph. According to Kaiser’s Rule, only factors with an eigenvalue exceeding unity should be retained. An alternative way is to look for an ‘elbow’ in the scree plot, i.e., the point after which the remaining factors decline in approximately a linear fashion, and to retain only the factors above the elbow. Finally, information criteria, such as the information criterion proposed by Bai and Ng (2002), can be used.

After deciding on the number of factors, it is possible that the factors of the (standardized) solution of the model are difficult to interpret. In that case, the factor loadings can be rotated yielding a solution that may be easier to interpret because the matrix has a simpler structure. Ideally, each indicator is correlated with as few factors as possible. The rotation technique that we use to interpret the factors is the oblimin rotation, which allows for correlation among the factors and minimizes the correlation of the columns of the factor loadings matrix. As a result, a typical indicator will have high factor loadings on one factor, while it has low loadings on the other factors.

All indicators receive factor scores for the various dimensions (factors) identified. These factor scores are used to come up with the so-called Bartlett predictor, i.e., the best linear unbiased predictor of the factor scores:

4 $$ \hat{\xi }_{i} = (\Updelta^{\prime } \Upomega^{ - 1} \Updelta )^{ - 1} \Updelta^{\prime } \Upomega^{ - 1} x_{i} $$

These factor scores can be used as a proxy for the latent variable.

## Factor Analysis: Results

### Political Factors

On the basis of the literature review of Sect. 2, we perform factor analysis on indicators of the type of regime, the stability of the regime and governance.^7^ Table 8 in the “Appendix” shows the countries included in the analysis. We only include countries if at least 80% of the required data is available. The number of countries included in the factor analysis ranges between 169 for the stability of the regime to 140 for governance. For some countries one or two indicators are not available for some country-years. We have less than 7% missing values. In order not to lose valuable information, we applied the EM algorithm of Dempster et al. (1977) to compute the missing observations.^8^ Table 1 shows the indicators, their sources, and the factor loadings for the three dimensions of the political regime in place to which we apply explanatory factor analysis.^9^ We use averages over the period 1980–1999 to make sure that the political variables employed in the model explaining cross-country differences in human capital (measured over the period 2000–2008) are exogenous. The detailed results of the factor analysis are presented in an appendix that is available on request (electronic supplementary material).

**Table 1:** Indicators of political institutions and their sources

Type of regime		Factor
Political rights	Freedom House	0.985**
Civil liberty	Freedom House	0.967**
Regulations of chief executive recruitment	Jaggers and Gurr (2006)	0.750**
Competition of chief executive selection	Update of Beck et al. (2001)	0.929**
Openness of chief executive	Update of Beck et al. (2001)	0.496**
Decision rules	Update of Beck et al. (2001)	0.943**
Competition of participation	Update of Beck et al. (2001)	0.942**
Way of election	Update of Beck et al. (2001)	0.622**
Executive competition	Databanks International (2005)	0.735**
Executive legitimacy	Databanks Internationall (2005)	0.868**
Type of regime	Databanks Internationall (2005)	0.360**
Parliamentary responsibility	Databanks Internationall (2005)	0.502**
Legislator selection	Databanks Internationall (2005)	0.427**
Military in politics	International Country Risk Guide (2003)	0.711**
Democratic accountability	International Country Risk Guide (2003)	0.891**

Political instability		Factor 1	Factor 2	Factor 3	Factor 4
Regime durability	Jaggers and Gurr (2006)	−0.143*	−0.031*	−0.366**	0.106*
Polarization	Update of Beck et al. (2001)	0.015	0.000	0.191*	0.564**
Political cohesion	Update of Beck et al. (2001)	−0.065	0.008	0.330**	0.661**
Government fractionalization	Update of Beck et al. (2001)	0.016	−0.071*	0.223**	0.493**
Number of assassinations	Databanks Internationall (2005)	0.569**	0.166**	−0.063*	0.220**
Number of strikes	Databanks Internationall (2005)	0.130*	0.664**	−0.103**	0.311**
Number of guerrilla	Databanks Internationall (2005)	0.997**	0.154**	−0.215*	−0.041*
Government crises	Databanks Internationall (2005)	0.231**	0.485**	0.285**	0.570**
Number of purges	Databanks Internationall (2005)	0.186**	0.167*	−0.271**	−0.118**
Number of riots	Databanks Internationall (2005)	0.189**	0.889**	−0.058*	−0.027*
Number of revolutions	Databanks Internationall (2005)	0.735**	0.063	0.541**	−0.121**
Number of anti-government demonstrations	Databanks Internationall (2005)	0.068*	0.906**	0.038*	0.075*
Coalitions	Databanks Internationall (2005)	0.100**	0.065	0.424**	0.355**
Number of coups	Databanks Internationall (2005)	0.130**	0.000	0.687**	−0.033*
Number of legislative elections	Databanks Internationall (2005)	−0.092*	0.181**	0.498**	0.258**
Number of executive elections	Databanks Internationall (2005)	0.122*	0.222**	0.734**	−0.075*
Number of constitutional changes	Databanks Internationall (2005)	0.031	0.459**	0.158*	0.653**
Number of cabinet changes	Databanks Internationall (2005)	0.084	0.318**	0.482**	0.378**
Government stability	International Country Risk Guide (2003)	−0.292**	−0.239**	−0.722**	−0.014
Internal conflict	International Country Risk Guide (2003)	−0.565**	−0.099	−0.812**	0.283*
External conflict	International Country Risk Guide (2003)	−0.386**	−0.041	−0.704**	0.365**
Ethnic tension	International Country Risk Guide (2003)	−0.409**	−0.274*	−0.715**	0.311**

Governance		Factor
Legislator effectiveness	Databanks Internationall (2005)	0.502**
Control of corruption	International Country Risk Guide (2003)	0.703**
Rule of law	International Country Risk Guide (2003)	0.859**
Bureaucratic quality	International Country Risk Guide (2003)	0.775**
Legal system and property rights	Gwartney and Lawson (2006)	0.918**
Regulation	Gwartney and Lawson (2006)	0.367**

** Significant at a 5% level, * significant at a 10% level

In the factor analysis on the type of regime (or democracy) we include indicators related to electoral rules, democratic accountability, and political freedom. The results of the factor analysis on democracy show that democracy is a highly significant (compared to a saturated model) one-dimensional construct, which explains more than 60% of the variance.

In the factor analysis on political instability, we include a number of indicators on the number of elections, polarization within the government, regime changes, civil aggression and protest. In line with the results of Jong-A-Pin (2009), we find four factors for political instability. The first factor is highly correlated with guerrilla, revolutions, and internal conflict and therefore we call this factor “aggression”. The second factor is highly correlated with strikes, riots, and anti-governmental demonstrations and therefore we call this factor “protest”. The third factor is highly correlated with number of coupes, regime durability, and constitutional changes and therefore we call this factor “regime instability”. The final factor is highly correlated with polarization and political cohesion and therefore we call this factor “government instability”. The correlation matrix of these four dimensions of political instability indicates that each factor measures a different dimension of political instability, because the correlations are very low. Together, these four factors explain about 60% of the variance.

Finally, in the factor analysis on governance, we use indicators on government effectiveness and regulation. The results of the factor analysis indicate that governance can be represented by a significant one-factor model, which explains about 70% of the variance.^10^

### Human Capital

To come up with a better measure for human capital that includes more information and to determine whether the human capital has a multidimensional character, we apply EFA to sixteen human capital indicators at the national level. The data used are averages over the period 2000–2008. First, we consider three indicators of education levels. Most previous studies on human capital used some indicator in this category to proxy the level of human capital in a country (cf. Benhabib and Spiegel 1994; Mankiw et al. 1992). A problem with these indicators is that they only account for formal education. We therefore include a second group of four indicators of skills that may be obtained by formal and informal education. As the concept of human capital is clearly much broader than education and skills, we also include three indicators of labor market experience.^11^ The more and longer people work, the more on the job training they arguably receive and hence the higher their human capital will be. Finally, we include five indicators of science and technological development. The more innovative a country is, the higher its human capital stock due to study, on the job training, and experience.

For some of the 123 countries one or two indicators are not available for some country-years. We have less than 4% missing values. In order not to lose valuable information, we applied the EM algorithm of Dempster et al. (1977) to compute the missing observations.

The results of the factor analysis in Table 2 show that human capital can be represented as a two-dimensional construct [detailed results are shown in an appendix that is available on request (electronic supplementary material)]. The two-factors model can explain about 88% of the number of people in R&D, but less than 20% of the percentage of high technological exports. In total about 70% of the variance is explained by the two factors, while 30% of the total variance is unique, meaning that this part is unexplained. Since the oblimin rotation minimizes the correlation between columns of the factor loadings matrix, the general pattern that arises is that most indicators have a high loading on one factor.

**Table 2:** Human capital indicators

	Source	Factor 1	Factor 2
Education			
Enrolment rate primary education	Cohen and Soto (2007)	0.285**	0.594**
Enrolment rate secondary education	Cohen and Soto (2007)	0.860**	−0.409**
Enrolment rate tertiary education	Cohen and Soto (2007)	0.894**	−0.188*
Skills			
Mathematics scores	Altinok and Murseli (2007)	0.893**	0.234**
Sciences scores	Altinok and Murseli (2007)	0.861**	0.274**
Reading scores	Altinok and Murseli (2007)	0.892**	0.282**
Literacy rate	World Bank (2009)	0.351**	0.703**
Labor market experience			
Years experience with primary education	Own calculations^a^	−0.053*	0.729**
Years experience with secondary education	Own calculations	0.769**	−0.231**
Years experience with tertiary education	Own calculations	0.802**	−0.143*
Average year of schooling	Barro and Lee (2010)	0.913**	−0.339**
Science and technology			
High technological export as % of GDP	World Bank (2009)	0.405**	−0.015*
Number of researchers in R&D	World Bank (2009)	0.888**	0.171**
Scientific and technical journal articles	World Bank (2009)	0.851**	0.217**
Number of technicians in R&D	World Bank (2009)	0.717**	0.133*
Number of patents per 1,000 people	World Bank (2009)	0.480**	0.106*

** Significant at a 5% level, * significant at a 10% level

^a^Computed on the basis of data from the World Bank (2009), Cohen and Soto (2007), and Barro and Lee (2001, 2010)

In the first factor the secondary and tertiary education indicators as well as the number of researchers, technicians, and scientific journal articles score high so we call this factor ‘advanced human capital’. In the second factor the primary education indicators scores high as well as the literacy rate, so we call this factor ‘basic human capital’. Pearson’s rank correlation coefficient between the two types of human capital is only 0.13 (although significant at the 10% level), showing that the two factors measure two different dimensions of human capital. Table 2 presents the indicators used, their sources, and loadings in the two-factors model, while the complete factor analysis is shown in an appendix that is available on request (electronic supplementary material).

## Structural Model: Method

To estimate the relationship between political factors and human capital, we use structural equation modeling. When the variables of interest are easily observable and can be measured without an error, regression analysis will generally suffice for the analysis of dependence. However, often the variables of interest are non-observable or latent (like human capital and political factors). Therefore a structural equation model should be used as a statistical technique to analyze the dimensions of a latent construct and analyze the dependence structure (see Dreher et al. 2007; Jöreskog 2000). A structural equation model is characterized by two basic components: (1) the measurement model, which allows using several variables (or indicators) for a single latent independent or dependent variable and (2) the structural model, which relates independent to dependent variables. The first part of the model is related to factor analysis, while the second part of the model can be compared to regression analysis. By combining these two analyses in one model, the measurement error is reduced and the reliability is increased.^12^

**Fig. 1:**
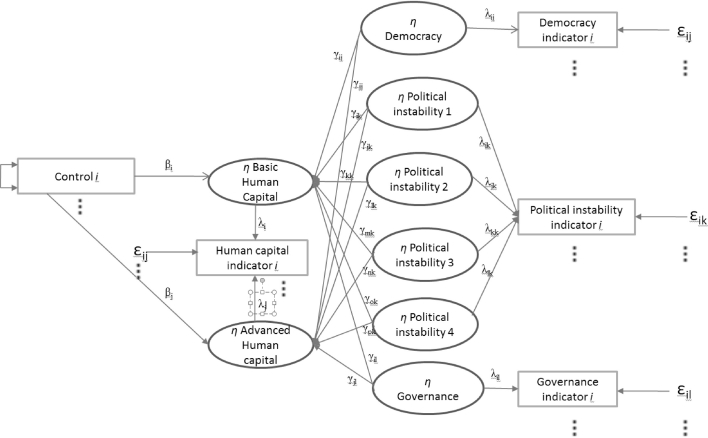
Path diagram structural equation model. Where *basic human capital* and *advanced human capital* are our unobserved measures for human capital

The structural part of the structural equation model is comparable with regression analysis and can be explained by a so-called path diagram. Figure 1 shows the path diagram for our model. The unobservable *η* variables are enclosed in circles and the observed variables are represented by rectangles. The error terms are represented by *ε*. The arrows leading from the observed variables to the latent variables indicate their hypothesized direct effect on the latent variables. The arrows leading from *η* to the various indicators represent the hypothesized impact of latent variables on the various indicators. The strength of the effects of variables is indicated by *λ*.

The vector *control* contains control variables that have been suggested in previous studies. These include the level^13^ and distribution of income,^14^ fertility rate (Becker et al. 1990), life expectancy, general government spending, public education spending, investment, foreign direct investment (Noorbakhsh et al. 2001), net migration (Haque and Kim 1994), highest marginal tax rate, unemployment, openness of trade, total population, age dependency rate, pupil–teacher ratio (Baum and Lake 2003), and rural population. Furthermore we include three variables that control for the information channels available in a country: number of people that have access to a newspaper, radio, or internet. Finally, we include the share of females in the total population. Table 9 in the “Appendix” provides an overview of all variables, their definition, as well as their sources. Like the political variables, the control variables are measured as averages over the period 1980–1999 (except initial income that is taken in 1980). The arrow from and to the *control* indicates that the various control variables may correlate. We add also country group effects into the model.^15^

## Structural Model: Results

The structural equation model with all control variables but without the political variables is taken as our starting point for the general to specific approach (see, for instance, Campos et al. 2004).^16^ That is, we estimate a model including all control variables (except the political variables). Next, we drop the least significant variable from the regression specification and estimate the model again. We repeat this procedure until only significant variables at a 10% level remain. We take the final result of the general to specific approach as our baseline model that is shown in columns (1) and (2) of Table 3.^17^ We allow for non-linearities in the relation between income and human capital. The reason is that at a certain level of development people tend not to invest anymore in ‘basic human capital’ but only in ‘advanced human capital’.

**Table 3:** Structural equation model estimation I

	Basic	Advanced	Basic	Advanced	Basic	Advanced	Basic	Advanced
	(1)	(2)	(3)	(4)	(5)	(6)	(7)	(8)
Initial income	2.799** [3.79]	0.416** [9.68]	2.951** [4.13]	0.352** [8.81]	2.517** [3.28]	0.331** [8.25]	3.042** [3.79]	0.340** [6.87]
Initial income squared	−0.318** [−4.55]		−0.331** [−4.13]		−0.225** [−4.47]		−0.338** [−5.26]	
Income inequality	1.618* [1.94]	−1.401** [−4.51]	1.466* [1.70]	−1.713** [−4.58]	1.422* [1.72]	−1.090** [−3.39]	1.342** [2.15]	−1.269** [−3.37]
Fertility rate	−1.143** [−2.48]		−1.185** [−2.54]		−0.920** [−2.21]		−1.256** [−2.41]	
Public educational spending	0.094** [1.99]	0.050** [1.99]	0.097** [2.13]	0.043** [1.78]	0.085* [1.87]	0.048** [2.26]	0.082** [2.02]	0.037* [1.73]
Life expectancy	0.198 [1.82]*		0.200* [1.81]		0.380 [1.57]		0.169** [2.00]	
Pupil–teacher ratio primary school	0.170* [1.84]		0.198* [1.76]		0.201* [2.20]		0.166* [1.77]	
Democracy			0.214** [2.01]	0.062 [0.82]				
Governance					0.028 [0.14]	0.272** [3.28]		
Instability: aggression							−0.031 [−0.39]	−0.029 [−0.79]
Instability: protest							−0.011 [−0.13]	−0.054 [−1.33]
Instability: regime							−0.187** [−2.23]	−0.098 [−1.52]
Instability: government instability							−0.011 [−0.13]	−0.181** [−2.27]
Number observations	123		111		105		112	
*R* ^2^	0.74		0.74		0.79		0.74	
χ^2^	2,772.75		2,578.41		2,686.67		2,734.71	
NFI	0.81		0.8		0.8		0.82	
CFI	0.82		0.81		0.8		0.85	

** Significant at a 5% level, * significant at a 10% level. The regressions are estimated with a constant and country group effect; *t* values are shown in brackets

The results indicate that the overall fit of the models is very good. The χ^2^ statistic, which compares the proposed model to an unrestricted alternative (saturated model), lies well below the 5% critical value. The norm fit index (NFI) and comparative fit index (CFI) range from zero to one, with values close to one indicating a better fit. The NFI has a value of about 0.81, while the CFI has a value of about 0.82. According to these measures, the model fits very well.

The level of income has a significant positive relationship with both types of human capital. For basic human capital we find a non-linear relationship: when income increases above about 8,000 US dollars (in prices of 2000), investment in basic human capital decreases.

More income inequality increases basic human capital, while it decreases advanced human capital. One explanation for this outcome is that when income is unequally distributed, a large share of the population is probably poor and does not have financial resources to invest in advanced human capital. Since income inequality and the human capital indicator are formulated as natural logs, we can interpret the estimated coefficients as elasticities. When income inequality increases by 1%, basic human capital rises by 1.6%, while advanced human capital declines by 1.4%. The fertility rate and life expectancy have a positive significant effect on basic human capital, while public education spending has a positive effect on both types of human capital. The impact of public spending on basic human capital is about two times larger than that on advanced human capital. Finally, we find a significant positive effect of the pupil–teacher ratio on basic human capital.

In the model shown in columns (3) and (4), our political regime indicator is added. The results show that democracy has a significant positive relationship with basic human capital, although the impact is quite small. If our findings represent a causal relationship, a topic to which we will return later, an increase in democracy of 1%, implies that basic human capital will increase by 0.21%. However, the relationship between democracy and advanced human capital is insignificant. So, our results only partially confirm the conclusion of Baum and Lake (2003), Lake and Baum (2001) and Feng (2003) who all found a positive relationship between human capital and democracy.

Next, we add our governance measure (columns 5 and 6). This variable has no significant relationship with basic human capital, but it has a significant relationship with advanced human capital (the elasticity is 0.27). These results confirm the findings of Ahrend (2002) who reports a significant negative relationship between corruption and secondary enrolment rates.

Finally, we include the four dimensions of political instability to the baseline regression (columns 7 and 8). Only regime instability has a significant relationship with basic human capital, while government stability is significantly linked to advanced human capital.^18^

## Discussion

### Sample Selection

In the regressions shown in Table 3, we assumed that the political institutions have a homogenous impact across countries. However, coefficients may differ across countries or country groups due to heterogeneity. For instance, Brown (1999) argues that the impact of democracy on enrollment washes out when countries develop. Therefore, we perform three sample robustness checks. First, we re-estimate the regressions (3)–(6) of Table 3 with the random sample method, replicating the regressions 1,000 times by estimating it with a randomly changing sample of countries covering 40% of the sample. The first part of Table 4 shows the random sample results. The results are in line with our previous findings. We still find a direct positive link between democracy and basic human capital and a negative one between regime instability and basic human capital, while government instability and governance have a negative, respectively positive, relationship with advanced human capital.

**Table 4:** Sample selection

	Random sample		Developing countries		Industrialized countries	
	Basic	Advanced	Basic	Advanced	Basic	Advanced
Democracy	0.215* [1.81]	0.065 [0.87]	0.232** [2.08]	0.071* [1.71]	0.157 [1.14]	0.054 [0.46]
Instability: aggression	0.031 [0.13]	0.295 [3.56]**	0.042 [0.15]	0.395 [1.58]	0.026 [0.08]	0.260 [5.28]
Instability: protest	−0.032 [−0.33]	−0.028 [−0.76]	−0.033 [−.50]	−0.040 [−0.90]	−0.026 [−0.31]	−0.021 [−0.88]
Instability: regime	−0.010** [−2.13]	−0.051 [−1.52]	−0.012** [−2.45]	−0.053 [−1.58]	−0.009 [−0.07]	−0.049 [−1.43]
Instability: government	−0.175 [−1.24]	−0.097** [−2.33]	−0.177 [−1.30]	−0.104 [−1.21]	−0.151 [−1.55]	−0.075** [−2.71]
Governance	−0.011 [−0.13]	−0.178** [−2.16]	−0.012 [−0.18]	−0.179 [−1.47]	−0.007 [−0.10]	−0.153** [−3.22]

** Indicates significance at a 5% level, and * means significance at a 10% level. Estimated including the control variables of Table 4; *t* values are shown in brackets

Second, we differentiate between developing and industrialized countries. The results show that for the sample of developing countries democracy has a significant relationship with human capital, but for industrialized countries the coefficient of our democracy indicator is not significant. We also find that regime instability is significantly related to basic human capital in developing countries, while government instability and governance only have a significant relationship with advanced human capital in industrialized countries.

Finally, the number of countries included in the factor analysis on human capital and political indicators differs from those included in the structural equation model. It is well known that countries tend to produce fewer data on key variables when they are less democratic. This implies that authoritarian states are likely to be underrepresented in cross-national studies—which can lead to overestimating the benefits of democracy (Ross 2006). To check whether our results are determined by a selection bias we perform two sensitivity tests. First, we estimate the missing control variables using the EM logarithm suggested by Dempster et al. (1977) to solve ML problems with missing data, and re-estimate the structural equation model. Again, we find that democracy and regime instability are significantly linked to basic human capital, while government instability and governance are significantly related with advanced human capital. Secondly, we re-estimate the factor analysis for human capital and political institutions including only the countries for which we have all the control variables after which we re-estimate the structural equation model. The results do not significantly differ from those reported in Table 3 (all results are available upon request).

### Indirect Effects

So far, we have examined the direct relationship between various dimensions of the political regime in place and human capital. However, our political variables may also have an indirect relationship with human capital, for example through their link with income. To test this hypothesis we add a regression for income to our model. In this model, we replace income in 1980 by average income between 1995 and 2000 in the regressions for human capital and again used the general-to-specific approach to formulate our model. In the regression for income, we use the secondary and primary school enrolment rates in the period 1980–1995 as regressors since most of the indicators used to construct our measures of human capital are often not available for this period. We employ ‘lagged’ human capital indicators in the regression for income in an attempt to avoid causality issues. Other variables that turn out to be significant in the income regression are population growth, and investment.

The first column of Table 5 shows our baseline model. The results for human capital are similar to the findings reported in Table 3. We find that secondary education, population growth, and investments are significantly related to income. When we add democracy, we do not only find a significant relationship between democracy and basic human capital, but also one between democracy and the level of income (columns 4–6). So democracy is also indirectly related to human capital. Similarly, governance is directly related to advanced human capital but has also an indirect relationship via its link with income. However, political instability has no indirect relationship with human capital via income.^19^

**Table 5:** Structural equation model taking the effect on income into account

	Basic	Advanced	Income	Basic	Advanced	Income	Basic	Advanced	Income	Basic	Advanced	Income
	(1)	(2)	(3)	(4)	(5)	(6)	(7)	(8)	(9)	(10)	(11)	(12)
Initial income	2.937** [4.94]	0.353** [7.88]		2.749** [3.59]	0.362** [8.63]		3.005** [3.51]	0.247** [6.65]		3.352** [4.64]	0.357** [7.54]	
Initial income squared	−0.316** [−4.24]			−0.337** [−4.62]			−0.298** [−3.66]			−0.326** [−5.11]		
Income inequality	1.719** [2.03]	−1.399** [−4.53]		1.609** [2.03]	−1.787** [−5.36]		1.521** [2.14]	−1.174** [−2.51]		1.349** [2.37]	−1.551** [−3.70]	
Fertility rate	−1.190** [−2.96]			−1.071** [−2.53]			−0.880* [−1.94]			−1.255** [−2.34]		
Public educational spending	0.100** [2.26]	0.049** [2.03]		0.088** [2.41]	0.045** [2.17]		0.086** [2.03]	0.051** [2.18]		0.079** [2.46]	0.037** [1.95]	
Life expectancy	0.206* [1.71]			0.206* [1.70]			0.327* [1.81]			0.348** [1.99]		
Pupil/teacher ratio	0.163** [2.24]			0.180* [1.81]			0.201* [1.79]			0.191** [2.17]		
Secondary education			0.868** [2.57]			0.835** [2.30]			0.938** [2.52]			0.878** [3.19]
Population growth			−0.320** [−2.52]			−0.351** [−2.52]			−0.328** [−2.06]			−0.313** [−2.34]
Investment			0.221* [1.94]			0.194** [2.28]			0.242** [2.42]			0.226* [1.80]
Democracy				0.193** [2.01]	0.058 [0.85]	0.554** [2.23]						
Governance							0.027 [0.13]	0.255** [3.13]	0.906** [2.43]			
Instability: aggression										−0.027 [−0.38]	−0.026 [−0.81]	−0.111 [−1.14]
Instability: protest										−0.009 [−0.12]	−0.049 [−1.23]	−0.019 [−0.22]
Instability: regime										−0.304** [2.36]	0.093 [1.62]	−0.488 [1.19]
Instability: government										−0.01 [−0.14]	−0.362** [−1.99]	−0.118 [1.20]
Number observations	102			101			95			102		
*R* ^2^	0.8			0.82			0.82			0.83		
χ^2^	3,084.78			3,335.3			3,234.3			3,248.36		
NFI	0.84			0.85			0.84			0.85		
CFI	0.85			0.86			0.85			0.85		

*T* statistics are shown in brackets. ** Significant at a 5% level, * significant at a 10% level. The regressions are estimated with a constant and country group effect

Finally, we also test the indirect relationship between our political variables and human capital through a possible link with income distribution, life expectancy, pupil–teacher ratio, and public education expenditure. However, when the indirect effect of income is included these other indirect effects are all insignificant. Also these results are available upon request. The level of democracy may influence political stability (or vice versa). Therefore we re-estimate the structural equation model including a relation running from democracy to political instability. This relation turns out to be insignificant for all dimensions of political instability. Also the relation running from the dimensions of political instability to democracy is not significant (results are available upon request).

### Method

Our results are based on structural equation modeling and in this respect our study differs from all previous research that we are aware of. So as a minimum, our results can be interpreted as an addition to the extant literature and a triangulation of the results reported so far. An important advantage of our approach is that it allows employing multiple indicators for each underlying latent construct such as human capital, democracy, governance and political instability. Furthermore, a structural equation model allows modeling rather complex interrelationships among various variables. At the same time, a disadvantage is that the method is exploratory rather than confirmatory. So, in general, causality is hard to establish. Of course, this is true for most cross-country studies. In an attempt to deal with this problem, we measure human capital over the period 2000–2008, while our political and control variables refer to the period 1980–1999. As it is highly unlikely that human capital over the period 2000–2008 affects our political variables as measured over 1980–1999, we interpret our findings as causal.

To check how sensitive our results are to the methodology used, we also estimate a cross-sectional regression model. Using the factor scores of human capital as our dependent variable and the factor scores on the political factors as explanatory variables. The estimated model is the following

5 $$ HC_{li} = \beta_{0} + \beta_{j} X_{ji} + \theta_{1} POLITICAL_{i} + \delta_{i} + \varepsilon_{i} $$

where *HC*_*li*_ is our measure for human capital of level *l* (advanced or basic) of country *i*. The vector *POLITICAL* contains our political variables, i.e., our indicators of the regime in place, governance and political instability found using the factor analysis*.* The vectors *X* contain *j* control variables. We include the same control variables as reported in Table 5. The variable *δ*_*i*_ represents country group fixed effects, while *ε*_*i*_ is the error term. As our dependent and main explanatory variables were obtained by estimation, we use bootstrap estimation to obtain consistent standard errors in our panel regression.

The results in the first part of Table 6 show that although the estimated coefficients differ from those reported in Table 3, which is probably caused by differences in optimization, we still find that democracy has a positive relationship with basic human capital, while regime instability has a negative relationship with basic human capital. Likewise, government instability has a negative relationship with advanced human capital. Finally we find that governance has a positive relation with advanced human capital.

**Table 6:** Cross-section and panel estimates

	Cross-section		Panel	
	Basic	Advanced	Basic	Advanced
Democracy	0.178** [1.98]	0.051 [0.52]	0.120** [2.02]	0.070 [0.76]
Governance	0.007 [0.52]	0.165** [3.12]	0.026 [0.14]	0.219** [2.88]
Instability: aggression	−0.017 [−0.98]	−0.023 [−1.01]	−0.024 [−0.45]	−0.037 [−0.68]
Instability: protest	−0.008 [−0.24]	−0.074 [−0.78]	−0.010 [−0.12]	−0.055 [−1.35]
Instability: regime	−0.213** [−1.97]	−0.078 [−1.32]	−0.151* [−1.90]	−0.049 [−1.54]
Instability: within regime	−0.029 [−0.34]	−0.198** [−1.97]	−0.013 [−0.10]	−0.106** [−2.03]

** Indicates significance at a 5% level, and * means significance at a 10% level. Estimated including the control variables of Table 5. *T* statistics are shown in brackets

As an additional robustness check, we also estimate a panel model. Gathering sufficient human capital data for earlier years for our large sample of countries turned out to be problematic. Therefore, the panel refers to the period 2000–2008. First, we perform factor analyses on our human capital and political indicators using annual data. The estimated model is:

6 $$ HC_{li,t} = \beta_{0} + \beta_{j} X_{j,i,t - 1} + \theta_{1} POLITICAL_{i,t - 1, \ldots ,t - 20} + \delta_{i} + \delta_{t} + \varepsilon_{i} $$

where *HC*_*li,t*_ is our measure for human capital of level *l* (advanced or basic) of country *i* at time *t* (*t* = 2000, 2001, …, 2008). The vector *POLITICAL* contains our political variables, i.e., our indicators of the regime in place, governance and political instability*.* These variables are measured as averages over the preceding 20 years. The vector *X* contains *j* control variables. We include the same control variables as reported in Table 5. The variables *δ*_*i*_ and *δ*_*t*_ represent country group and time fixed effects, while *ε*_*t*_ is the error term.

The results, as shown in the second part of Table 6, confirm the results from the first part of Tables 3 and 6. We therefore conclude that our results are not driven by our choice for structural equation modeling. As a further check on the sensitivity of our findings, we re-estimate the panel model using political variables defined as averages over the proceeding 10 and 5 years, respectively. The qualitative results (available on request) are similar to those reported in Table 6.

### Reverse Causality

According to Castello-Climent (2008) and Acemoglu et al. (2005), there exists also a relation running from human capital to democracy. In this section we test for this relation. We use the primary enrolment rate between 1960 and 1979 as an indicator of basic human capital and tertiary education during this period as a proxy for advanced human capital, as many other indicators that we used to construct our preferred measures of human capital are not available for earlier years.

Our dependent political variables (democracy, political instability and governance) are taken as an average of the period 1980–1999. The remaining control variables are based on Castello-Climent (2008) and Acemoglu et al. (2005) and include the level and distribution of income, life expectancy, and total population. The results as shown in Table 7 indicate that the primary education enrolment rate is not a significant determinant of political institutions. However, we find that the secondary education enrolment rate has a significant relationship with the level of democracy and governance within a country. This result confirms the conclusion of earlier studies by Castello-Climent (2008) and Acemoglu et al. (2005).

**Table 7:** Reverse causality

	Democracy	Aggression	Protest	Regime instability	Government instability	Governance
Income	0.318** [1.99]	−0.307* [−1.69]	−0.378* [−1.88]	−0.313** [−1.98]	−0.352** [−2.00]	0.260** [2.13]
Life expectancy ratio	0.176 [1.33]	−0.186 [−1.50]	−0.208 [−1.39]	−0.194 [−1.30]	−0.144 [−1.20]	0.149 [1.37]
Total population	−0.197 [−1.32]	0.184 [1.26]	0.217 [1.15]	0.213 [1.48]	0.164 [1.25]	−0.194 [−1.26]
Income equality	0.618* [1.78]	−0.545* [−1.85]	−0.720** [−2.04]	−0.597 [−1.62]	−0.503** [−2.02]	0.668* [1.66]
Secondary education enrolment rate	0.165** [2.01]	−0.138 [−1.52]	−0.187 [−1.46]	−0.184 [−1.29]	−0.177 [−1.25]	0.178** [2.78]
Primary education enrolment rate	0.089 [1.15]	−0.103 [−1.29]	−0.087 [−1.02]	−0.105 [−1.04]	−0.094 [−1.03]	0.086 [1.11]

** Indicates significance at a 5% level, and * means significance at a 10% level. *T* statistics are shown in brackets

## Conclusions

The purpose of this paper is to examine whether political institutions affect the accumulation of human capital. This is an important issue, as several studies conclude that human capital is one of the main drivers of economic growth. Previous studies report a positive significant impact of some proxy for democracy on various human capital indicators. However, most studies analyzing the impact of political institutions on cross-country differences in human capital have a measurement problem. Most studies employ school enrolment rates or average years of schooling as an indicator of human capital, thereby implicitly assuming that human capital is a one-dimensional concept. Likewise, political indicators are frequently chosen in a rather arbitrary way.

To overcome these measurement problems, we examine the relationship between different dimensions of the political regime in place and human capital using a two-step structural equation model. In the first step, we employ factor analysis on 16 human capital indicators to construct new human capital measures. It turns out that a two-factor model captures most of the variance of the various indicators. On the basis of this finding, we constructed two variables: basic human capital and advanced human capital.

To construct measures of political institutions, we applied factor analysis on 3 sets of political system indicators, i.e., the regime in place, political instability, and governance. It turns out that democracy and governance can be represented by a one-dimensional construct and that political instability is a four-dimensional construct.

In the second step, we analyse the impact of our political variables on human capital, using a cross-sectional structural model for some 100 countries including various economic and demographic control variables. We use the general-to-specific approach to decide on the specification of our model. We conclude that democracy is positively related to basic human capital, while regime instability has a negative link with basic human capital. Governance has a positive relationship with advanced human capital, while government instability has a negative link with advanced human capital. Finally, we also find an indirect positive effect of governance and democracy on both types of human capital through their effect on income.

We also check whether our findings are robust for our preferred modeling approach by also estimating cross-section and panel models. The results are in line with those of the structural equation model.

### Electronic supplementary material

Below is the link to the electronic supplementary material.

Supplementary material 1 (DOC 147 kb)

## Appendix

See Tables 8 and 9.

**Table 8:** Countries included in the various factor analyses

	Country	Human capital	Governance	Democracy	Political instability
1	Afghanistan	●		●	●
2	Albania	●		●	●
3	Algeria		●	●	●
4	Angola		●	●	●
5	Argentina	●	●	●	●
6	Armenia		●	●	●
7	Australia	●	●	●	●
8	Austria	●	●	●	●
9	Azerbaijan		●	●	●
10	Bahamas		●		●
11	Bahrain	●	●	●	●
12	Bangladesh	●	●	●	●
13	Barbados	●	●	●	●
14	Belarus		●	●	●
15	Belgium	●	●	●	●
16	Belize				●
17	Benin	●		●	●
18	Bhutan			●	●
19	Bolivia	●	●	●	●
20	Bosnia-Herzegovina			●	●
21	Botswana	●	●	●	●
22	Brazil	●	●	●	●
23	Brunei	●	●		●
24	Bulgaria	●	●	●	●
25	Burkina Faso	●	●	●	●
26	Burma		●	●	●
27	Burundi			●	●
28	Cambodia			●	●
29	Cameroon	●	●	●	●
30	Canada	●	●	●	●
31	Cape Verde	●			●
32	Central African Republic	●		●	●
33	Chad			●	●
34	Chile	●	●	●	●
35	China	●	●	●	●
36	Colombia	●	●	●	●
37	Comoros			●	●
38	Congo (Brazzaville)	●	●	●	●
39	Congo (Kinshasa)	●	●	●	●
40	Costa Rica	●	●	●	●
41	Cote d’Ivoire		●	●	●
42	Croatia	●	●	●	●
43	Cuba		●	●	●
44	Cyprus	●	●	●	●
45	Czech Republic	●	●	●	●
46	Denmark	●	●	●	●
47	Djibouti			●	●
48	Dominica				●
49	Dominican Republic	●	●	●	●
50	Ecuador	●	●	●	●
51	Egypt, Arab Rep.	●	●	●	●
52	El Salvador	●	●	●	●
53	Equatorial Guinea			●	●
54	Eritrea			●	●
55	Estonia	●	●	●	●
56	Ethiopia		●	●	●
57	Fiji	●		●	●
58	Finland	●	●	●	●
59	France	●	●	●	●
60	Gabon		●	●	●
61	Gambia, The	●	●	●	●
62	Georgia			●	●
63	Germany	●	●	●	●
64	Ghana	●	●	●	●
65	Greece	●	●	●	●
66	Guatemala	●	●	●	●
67	Guinea		●	●	●
68	Guinea-Bissau		●	●	●
69	Guyana	●	●	●	●
70	Haiti	●	●	●	●
71	Honduras	●	●	●	●
72	Hong Kong	●	●		
73	Hungary	●	●	●	●
74	Iceland	●	●	●	●
75	India	●	●	●	●
76	Indonesia	●	●	●	●
77	Iran	●	●	●	●
78	Iraq	●	●	●	●
79	Ireland	●	●	●	●
80	Israel	●	●	●	●
81	Italy	●	●	●	●
82	Jamaica	●	●	●	●
83	Japan	●	●	●	●
84	Jordan	●	●	●	●
85	Kazakhstan	●	●	●	●
86	Kenya	●	●	●	●
87	Korea, South	●	●	●	●
88	Kuwait	●	●	●	●
89	Kyrgyzstan			●	●
90	Laos			●	●
91	Latvia	●	●	●	●
92	Lebanon		●		●
93	Lesotho	●	●	●	●
94	Liberia	●		●	●
95	Libya		●	●	●
96	Lithuania	●	●	●	●
97	Luxembourg	●	●	●	●
98	Macedonia	●		●	●
99	Madagascar	●	●	●	●
100	Malawi	●	●	●	●
101	Malaysia	●	●	●	●
102	Mali	●	●	●	●
103	Malta	●	●		●
104	Mauritania			●	●
105	Mauritius	●		●	●
106	Mexico	●	●	●	●
107	Moldova		●	●	●
108	Mongolia		●	●	●
109	Morocco		●	●	●
110	Mozambique	●	●	●	●
111	Namibia	●	●	●	●
112	Nepal	●		●	●
113	The Netherlands	●	●	●	●
114	New Zealand	●	●	●	●
115	Nicaragua	●	●	●	●
116	Niger	●	●	●	●
117	Nigeria		●	●	●
118	North Korea		●	●	●
119	Norway	●	●	●	●
120	Oman		●	●	●
121	Pakistan	●	●	●	●
122	Panama	●	●	●	●
123	Papua New Guinea	●	●	●	●
124	Paraguay	●	●	●	●
125	Peru	●	●	●	●
126	Philippines	●	●	●	●
127	Poland	●	●	●	●
128	Portugal	●	●	●	●
129	Qatar		●	●	●
130	Romania	●	●	●	●
131	Russia	●	●	●	●
132	Rwanda	●		●	●
133	Saudi Arabia		●	●	●
134	Senegal	●	●	●	●
135	Serbia and Montenegro	●	●		●
136	Seychelles	●			
137	Sierra Leone	●	●	●	●
138	Singapore	●	●	●	●
139	Slovak Republic	●	●	●	●
140	Slovenia	●	●	●	●
141	Solomon Islands		●	●	●
142	Somalia			●	
143	South Africa	●	●	●	●
144	Spain	●	●	●	●
145	Sri Lanka	●	●	●	●
146	Sudan	●	●	●	●
147	Suriname		●		●
148	Swaziland	●		●	●
149	Sweden	●	●	●	●
150	Switzerland	●	●	●	●
151	Syria	●	●	●	●
152	Taiwan		●	●	●
153	Tajikistan			●	●
154	Tanzania		●	●	●
155	Thailand	●	●	●	●
156	Togo	●	●	●	●
157	Trinidad and Tobago	●	●	●	●
158	Tunisia	●	●	●	●
159	Turkey	●	●	●	●
160	Turkmenistan			●	●
161	Uganda	●	●	●	●
162	Ukraine		●	●	●
163	United Arab Emirates		●	●	●
164	United Kingdom	●	●	●	●
165	United States	●	●	●	●
166	Uruguay	●	●	●	●
167	Uzbekistan			●	●
168	Venezuela	●	●	●	●
169	Vietnam	●	●	●	●
170	Yemen		●	●	●
171	Zambia	●	●	●	●
172	Zimbabwe	●	●	●	●
	Number of countries	123	140	161	169

**Table 9:** Data used in regressions

Variable	Definition	Source
Age dependency ratio	The ratio of people younger than 15 or older than 64 to those aged 15–64	World Bank (2009)
Females	Share of females in the population	World Bank (2009)
Fertility rate	The number of children of a woman if she were to live to the end of her childbearing years and bear children in accordance with current age-specific fertility rates	World Bank (2009)
Foreign direct investment	The net inflows of investment to acquire a lasting management interest in an enterprise operating in an economy other than that of the investor as a share of GDP	World Bank (2009)
Government spending	All government current expenditures, excluding publication spending, for purchases of goods and services as a share of GDP. Data are in constant 2,000 U.S. dollars	World Bank (2009)
Enrolment rate secondary education	Gross enrolment rate of secondary education	Cohen and Soto (2007)
Enrolment rate primary education	Gross enrolment rate of primary education	Cohen and Soto (2007)
Highest marginal tax rate	The highest marginal tax rate as applied to the taxable income of individuals	World Bank (2009)
Income inequality	Estimated household income inequality data set (EHII)—is a global dataset, derived from the econometric relationship between UTIP-UNIDO, other conditioning variables, and the World Bank’s Deininger and Squire data set	The University of Texas Inequality Project (2006)
Initial income	The sum of gross value added by all resident producers in the economy plus any product taxes and minus any subsidies not included in the value of the products on 1980. Data are in constant 2,000 U.S. dollars	World Bank (2009)
International migration	People born in a country other than that in which they live as % of population. It also includes refugees	World Bank (2009)
Internet per capita	Number of internet connections per capita	World Bank (2009)
Investment as % GDP	Includes land improvements; plant, machinery, and equipment purchases; and the construction of roads, railways, and the like, including schools, offices, hospitals, private residential dwellings, and commercial and industrial buildings. Data as share of GDP	World Bank (2009)
Life expectance	The number of years a newborn infant would live if prevailing patterns of mortality at the time of its birth were to stay the same throughout its life	World Bank (2009)
Newspaper per capita	Number of newspapers subscriptions per capita	World Bank (2009)
Radios per capita	Number of radios per capita	World Bank (2009)
Population	All residents regardless of legal status or citizenship—except for refugees not permanently settled in the country of asylum, who are generally considered part of the population of their country of origin	World Bank (2009)
Public educational spending	Current and capital public expenditure on education plus subsidies to private education at the primary, secondary, and tertiary levels	World Bank (2009)
Pupil–teacher ratio	Primary school pupil–teacher ratio is the number of pupils enrolled in primary school divided by the number of primary school teachers (regardless of their teaching assignment)	World Bank (2009)
Rural population	Rural population is calculated as the difference between the total population and the urban population	World Bank (2009)
Trade openness	The sum of exports and imports of goods and services measured as percentage of GDP	World Bank (2009)
Unemployment	The share of the labor force that is without work but available for and seeking employment	World Bank (2009)

## Footnotes

1 As Adams-Kane and Lim (2009) point out, there is a strong theoretical case that human capital can drive growth in both neoclassical and endogenous growth models, although there is also the possibility of reverse causality (Bils and Klenow 2000). Using panel data from 120 developing countries from 1975 to 2000, Baldacci et al. (2008) explore the direct and indirect channels linking social spending, human capital, and growth in a system of equations. These authors find that both education and health spending have a positive and significant direct impact on the accumulation of education and health capital, and thus can lead to higher economic growth. However, some papers do not report that human capital is significantly related to economic growth (see, for instance, Pritchett 2001). Recently, Sunde and Vischer (2011) replicated earlier results from the literature showing that both initial levels and changes in human capital have positive growth effects. They also report that the effects are heterogeneous across countries with different levels of development.

2 The same holds for most studies explaining cross-country growth differences. See Wößmann (2000) for a critical discussion of human capital measures used in growth regressions.

3 See also Pinto and Timmons (2005), Becker and Tomes (1979), Aghion and Bolton (1990), Castello-Climent and Domenech (2008), and Saint-Paul and Verdier (1993).

4 The first step in this analysis is to check whether the data used is suitable for an EFA using the Kaiser–Meyer–Olkin measure of sampling adequacy testing whether the partial correlation among variables is low. A test statistic that is higher than 0.6 indicates that the data is suitable for an EFA (Kaiser 1970). An alternative test is Bartlett’s test of sphericity, that checks whether the correlation matrix is an identity matrix in which case the factor model is inappropriate. Both tests indicate that the human capital data and political data used in the present paper are suitable for an EFA (Lattin et al. 2003).

5
*E*(*ε*) = 0 and *E*(ζ*ε′*) = 0.

6 We tested each specification for multivariate normality. In those cases where transformations have been necessary in order for the data to satisfy the multivariate normality assumption, the non-transformed data produced similar results.

7 Various indicators of governance also include information on the decision-making process within government. As this dimension is captured by our regime measure, we do not include it here.

8 The EM algorithm is an iterative method, which involves forming a log-likelihood function for the latent data as if they were observed and taking its expectation, while in the maximization step the resulting expected log-likelihood is maximized.

9 Arguably, democracy and governance are closely related. The correlation between the two is about 0.6. We performed a factor analysis on all indicators of governance and democracy. However, the results did not make much sense. This is also the case if we perform a factor analysis on all political indicators.

10 See also Klomp and De Haan (2008).

11 Experience is calculated as follows [share of labour force with education level *i*] × [1 − unemployment rate of education level *i*] × [average age of labour with education level *i* − years of education].

12 Using the factor scores as a regressor in a regression model would lead to inconsistent estimation results because the latent variables are imperfectly measured. In particular, the coefficients of the latent variables will be biased and inconsistent (Wansbeek and Meijer 2000; Bollen and Lennox 1991).

13 Causality between income and human capital may run in both directions (Narayan and Smith 2004). In Sect. 6 we will examine this in more detail.

14 Checchi (2000) reports a negative effect of income inequality on secondary education enrolment rates. However, Castello-Climent and Domenech (2008) find an insignificant result for income inequality when accounting for life expectancy.

15 We identified the following country groups: Western Europe, Eastern Europe, North and Central Asia, South Asia, Middle East, North America, Central America and the Caribbean, Latin America, North and Central Africa, Southern Africa, Australia and Oceania.

16 If we directly include our political variables into the general-to-specific method, our main findings do not change. Results are available on request.

17 The model is estimated with AMOS 7.0 using the maximum likelihood estimation.

18 We also included all six dimensions of the political system in one model. However, the results do not change substantially. The results are available on request.

19 The effect of democracy and governance on income is higher in developing countries. The indirect effects are confirmed by the outcomes of a bootstrapping analysis and rolling regressions. The results are available upon request.
