# Supplementary material for: Political Regime and Human Capital: A Cross-Country Analysis
Source: Soc Indic Res. 2012 Mar 20;111(1):45–73. doi: 10.1007/s11205-011-9983-6 (PMC3560949; doi:10.1007/s11205-011-9983-6)
Supplement: Supplementary file 1 — Supplementary material 1 (DOC 147 kb) [file 11205_2011_9983_MOESM1_ESM.doc]

**APPENDIX. Detailed results EFA (not for publication)**

**Factor analysis human capital**

The data used is an average over the period 2000 to 2008 for 123 countries. We have less than 4 percent missing values. In order not to lose valuable information, we applied the EM algorithm of Dempster *et al*. (1977) to compute the missing observations. The EM algorithm was suggested by Dempster *et al*. to solve maximum likelihood problems with missing data. It is an iterative method, the expectation step involves forming a log-likelihood function for the latent data as if they were observed and taking its expectation, while in the maximization step the resulting expected log-likelihood is maximized.

As can be seen from Table B1, the correlations between the different indicators are not perfect. Therefore, we consider the different indicators of individual human capital as imperfect measures of this concept.

To extract the right number of factors out of the various indicators, the scree plot is used (see Figure B1). Both the Kaiser rule and the elbow criteria indicate that two factors should be identified. The test-statistic of the two factor model Likelihood ratio test is 598.61 which is χ^2^(89) distributed and is highly significant at the five percent significance level, suggesting that the two-factor model is appropriate. This is confirmed by the Bai and Ng information test that is highly significant.

Table B2 presents the factor loading of the various indicators and the variance of the indicators explained by the two factors. The two-factors model can explain about 88 percent of the number of people in R&D, but less than 20 percent of the percentage of high technological exports. In total about seventy percent of the variance is explained by the two factors, while thirty percent of the total variance is unique, meaning that this part is unexplained.

Since the oblimin rotation minimizes the correlation between columns of the factor loadings matrix, the general pattern that arises is that every indicator has most time a high loading in one factor. On the basis of these results we can name these factors. In the first factor the secondary and tertiary education indicators as well as the number of researchers, technicians and journal articles score high so we call this factor ‘advanced human capital’. In the second factor the primary education indicators score high so we call this factor ‘basic human capital’. The correlation between the two types of human capital is 0.13. This is quit low, meaning that the two factors measure two different types of human capital.

Table B1. Correlation between human capital indicators

*** Significant at a 5 percent level, * significant at a 10 percent level.*

Figure B1. Scree plot of the eigenvalues and number of factors for the human capital indicators

Table B2. Factor matrix human capital

| **Human capital** | | | |
| --- | --- | --- | --- |
| Indicator | Factor 1 | Factor 2 | Variance explained |
| Enrolment rate primary education | 0.285 | -0.594 | 0.396 |
| Enrolment rate secondary education | 0.860 | -0.409 | 0.827 |
| Enrolment rate tertiary education | 0.894 | -0.188 | 0.803 |
| Literacy rate | 0.351 | 0.703 | 0.617 |
| Mathematics score | 0.893 | 0.334 | 0.844 |
| Sciences score | 0.861 | 0.374 | 0.810 |
| Reading score | 0.892 | 0.282 | 0.822 |
| Labor force with primary education | -0.053 | 0.729 | 0.554 |
| Labor force with secondary education | 0.769 | -0.231 | 0.608 |
| Labor force with tertiary education | 0.802 | -0.143 | 0.644 |
| Average years of schooling | 0.913 | -0.339 | 0.881 |
| High technological export as % of GDP | 0.405 | -0.015 | 0.165 |
| Number of researchers in R&D | 0.888 | 0.171 | 0.875 |
| Scientific and technical journal articles | 0.851 | 0.217 | 0.835 |
| Number of Technicians in R&D | 0.717 | 0.133 | 0.568 |
| Number of patents per 1000 people | 0.480 | 0.106 | 0.260 |
| Kaiser-Mayer-Olkin test | 0.693 |  |  |
| Bai and Ng test p-value | 0.000 |  |  |
| Likelihood ratio test p-value | 0.000 |  |  |

**Factor analysis political institutions**

*Type of the regime*

We start by employing factor analysis on the type of regime in place or democracy. For the factor analysis we use the mean of 15 indicators for the period 1989 to 1999 for 161 countries. The indicators relate to electoral rules, democratic accountability, and political freedom.

Figure B2 shows the scree plot. According to the Kaiser rule, more than three factors should be retained in both factor analyses. However, this is a so-called Heywood case where some solutions of the unique variances of the indicators are smaller than zero[[1]](#footnote-2). In general, a Heywood case is an indication of a poorly specified model. If instead the elbow criterion is used, democracy can in both cases be represented as a one-dimensional construct. The Likelihood Ratio test statistic of the one-factor model is 777.90 which is χ^2^(90) distributed and highly significant at a five percent level. The test rejects the null hypothesis that the estimate of a satured model is equal in favour of the restricted one-factor model. The factor loadings of the different indicators and the variance explained are shown in Table B1. In the static factor analysis the one-factor model can almost explain ninety percent of the variance of the civil and political rights indicators of Freedom House, while it only explains about ten percent of the regime type indicator of Databanks’ International. Overall, the one-factor model explains about 60 percent of the total variance.

Figure B2. Scree plot of the eigenvalue and factors of democracy


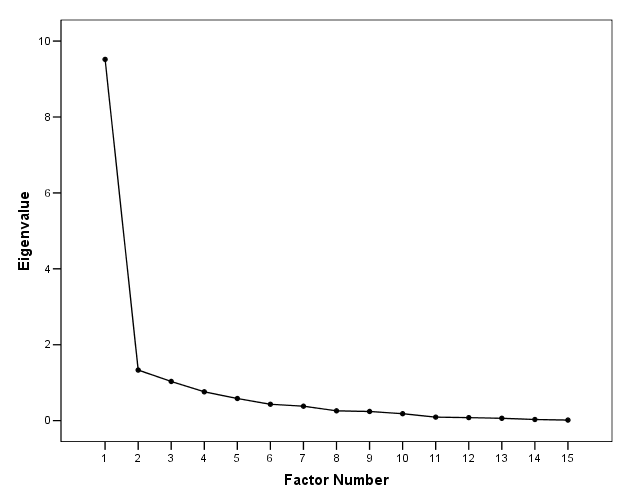


Table B3. Factor matrix democracy

| Indicator | Factor loadings | Variance explained |
| --- | --- | --- |
| Political rights | 0.985 | 0.970 |
| Civil liberty | 0.967 | 0.935 |
| Regulations of Chief Executive recruitment | 0.750 | 0.563 |
| Competition of Chief Executive selection | 0.929 | 0.862 |
| Openness of Chief Executive | 0.496 | 0.246 |
| Decision rules | 0.943 | 0.890 |
| Competition of participation | 0.942 | 0.887 |
| Way of election | 0.622 | 0.387 |
| Executive competition | 0.735 | 0.540 |
| Executive legitimacy | 0.868 | 0.754 |
| Type of regime | 0.360 | 0.129 |
| Parliamentary responsibility | 0.502 | 0.252 |
| Legislator selection | 0.427 | 0.183 |
| Military in politics | 0.711 | 0.505 |
| Democratic accountability | 0.891 | 0.794 |
| Kaiser-Mayer-Olkin test | 0.604 |  |
| Bai and Ng test p-value | 0.000 |  |
| Likelihood test p-value | 0.000 |  |

*Political instability*

For the analysis of indicators of the stability of the regime or political instability we follow the same procedure as Jong-A-Pin (2009). Compared to Jong-A-Pin (2009), we reduced the number of indicators included to raise the number of countries included. However, we confirm the main conclusions of Jong-A-Pin (2009). For the static analysis we use the mean 22 of political indicators for 169 countries between 1989 and 1999. The indicators include variables on the number of elections, polarization within the government, regime changes, civil aggression and protest.

Figure B3 shows the scree plot. We face the same problem as with democracy. According to the Kaiser rule, six factors should be extracted, but this is probably also a Heywood case. Following the ‘elbow criterion’, we can identify four factors in the scree plot, hence we decided to use the four factor model. The Likelihood Ratio statistic is 465.07, which is χ^2^(149) distributed and is significant at a five percent level. The factor loadings of the rotated factors are shown in Table B4. Overall, the four-factors model explains about sixty percent of the variance.

Since the oblimin rotation minimizes the correlation between columns of the factor loadings matrix, the general pattern that arises is that most indicators have a high loading on one factor. On the basis of these results we can therefore interpret the factors identified. The first factor is highly correlated with guerrilla, revolutions, and internal conflict and therefore we call this factor ‘aggression’. The second factor is highly correlated with strikes, riots, and anti-governmental demonstrations and therefore we call this factor ‘protest’. The third factor is highly correlated with number of coupes, regime durability, and constitutional changes and therefore we call this factor ‘regime instability’. The final factor is highly correlated with polarization and political cohesion and therefore we call this factor ‘government instability’. The correlation matrix of these four dimensions of political instability, as shown in Table B5, indicates that each factor measures a different dimension of political instability, because the correlations are very low.

# **Figure B3. Scree plot of the eigenvalue and factors of political instability**


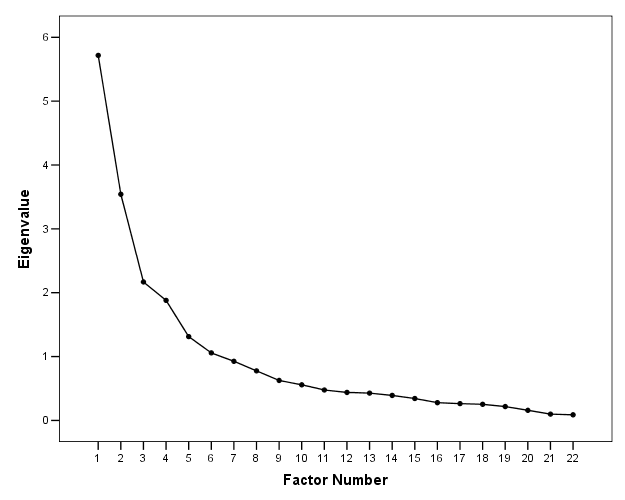


Table B4. Factor matrix political instability

| Indicator | Factor 1: aggression | Factor 2: protest | Factor 3: regime instability | Factor 4:  Within regime instability | Variance explained |
| --- | --- | --- | --- | --- | --- |
| Regime durability | -0.143 | -0.031 | -0.366 | 0.106 | 0.140 |
| Polarization | 0.015 | 0.000 | 0.191 | 0.564 | 0.335 |
| Political cohesion | -0.065 | 0.008 | 0.330 | 0.661 | 0.490 |
| Government fractionalization | 0.016 | -0.071 | 0.223 | 0.493 | 0.284 |
| Number of assassinations | 0.569 | 0.166 | -0.063 | 0.220 | 0.375 |
| Number of strikes | 0.130 | 0.664 | -0.103 | 0.311 | 0.485 |
| Guerrilla | 0.997 | 0.154 | -0.215 | -0.041 | 0.999 |
| Number of government crises | 0.231 | 0.485 | 0.285 | 0.570 | 0.594 |
| Number of purges | 0.186 | 0.167 | -0.271 | -0.118 | 0.112 |
| Number of riots | 0.189 | 0.889 | -0.058 | -0.027 | 0.842 |
| Number of revolutions | 0.735 | 0.063 | 0.541 | -0.121 | 0.690 |
| Number of Anti-government demonstrations | 0.068 | 0.906 | 0.038 | 0.075 | 0.843 |
| Coalitions | 0.100 | 0.065 | 0.424 | 0.355 | 0.304 |
| Number of coupes | 0.130 | 0.000 | 0.687 | -0.033 | 0.496 |
| Number of legislative elections | -0.092 | 0.181 | 0.498 | 0.258 | 0.331 |
| Number of executive elections | 0.122 | 0.222 | 0.734 | -0.075 | 0.557 |
| Number of constitutional changes | 0.031 | 0.459 | 0.158 | 0.653 | 0.481 |
| Number of cabinet changes | 0.084 | 0.318 | 0.482 | 0.378 | 0.563 |
| Government stability | -0.292 | -0.239 | -0.722 | -0.014 | 0.833 |
| Internal conflict | -0.565 | -0.099 | -0.812 | 0.283 | 0.615 |
| External conflict | -0.386 | -0.041 | -0.704 | 0.365 | 0.646 |
| Ethnic tension | -0.409 | -0.274 | -0.715 | 0.311 | 0.603 |
| Kaiser-Mayer-Olkin test | 0.693 |  |  |  |  |
| Bai and Ng test p-value | 0.000 |  |  |  |  |
| Likelihood ratio test p-value | 0.000 |  |  |  |  |

# Table B5. Correlation matrix factors

|  | Aggression | Protest | Regime instability | Within regime instability |
| --- | --- | --- | --- | --- |
| Aggression | 1.00 | 0.15 | 0.24 | 0.007 |
| Protest |  | 1.00 | 0.15 | 0.17 |
| Regime instability |  |  | 1.00 | 0.17 |
| Within regime instability |  |  |  | 1.00 |

*Governance*

The final political dimension to which we apply EFA is governance. For this analysis we used 6 indicators for 140 countries. On the basis of the Kaiser rule and the elbow criteria we decided to retain one factor. The Likelihood Ratio test statistic is 39.90, which is χ^2^(9) distributed and is highly significant at a five percent significance level. The factor loadings of the individual indicators and the explained variance of the individual indicators are shown in Table B6. It explains almost all variance of the legal system and property right index, while it explains less then fifteen percent of the regulation index. Overall, the model explains about seventy percent of the total variance.

Figure B4. Eigenvalues and factors of governance

Table B6. Factor matrix governance

| Indicator | Factor loadings | Variance explained |
| --- | --- | --- |
| Legislator effectiveness | 0.502 | 0.252 |
| Control of corruption | 0.703 | 0.494 |
| Rule of law | 0.859 | 0.738 |
| Bureaucratic quality | 0.775 | 0.601 |
| Legal system and property rights | 0.918 | 0.843 |
| Regulation | 0.367 | 0.135 |
| Kaiser-Mayer-Olkin test | 0.744 |  |
| Bai and Ng test p-value | 0.000 |  |
| Likelihood test p-value | 0.000 |  |

1. See Heywood (1931). [↑](#footnote-ref-2)
